# Multimerizations, Aggregation, and Transfer Reactions of Small Numbers of Molecules

**DOI:** 10.1021/acs.jcim.3c00774

**Published:** 2023-07-11

**Authors:** Ronen Zangi

**Affiliations:** † Donostia International Physics Center (DIPC), 20018 Donostia-San Sebastián, Spain; ‡ Department of Organic Chemistry I, University of the Basque Country UPV/EHU, 20018 Donostia-San Sebastián, Spain; § IKERBASQUE, Basque Foundation for Science, 48009 Bilbao, Spain

## Abstract

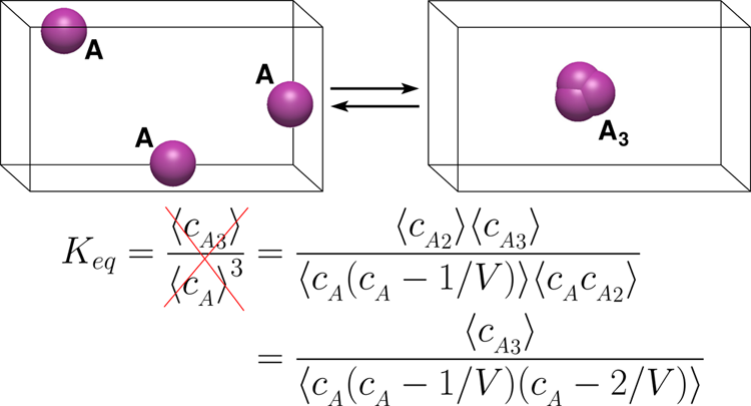

Chemical equilibria of multimerizations in systems with small numbers of particles exhibit a behavior seemingly at odds with that observed macroscopically. In this paper, we apply the recently proposed expression of equilibrium constant for binding, which includes cross-correlations in reactants’ concentrations, to write an equilibrium constant for the formation of clusters larger than two (e.g., trimer, tetramer, and pentamer) as series of two-body reactions. Results obtained by molecular dynamics simulations demonstrate that the value of this expression is constant for all concentrations and system sizes, as well as at an onset of a phase transition to an aggregated state, where densities in the system change discontinuously. In contrast, the value of the commonly utilized expression of equilibrium constant, which ignores correlations, is not constant and its variations can reach few orders of magnitude. Considering different paths for the same multimer formation, with elementary reactions of any order, yields different expressions for the equilibrium constant, yet, with exactly the same value. This is also true for routes with essentially zero probability to occur. Existence of different expressions for the same equilibrium constant imposes equalities between averages of correlated, along with uncorrelated, concentrations of participating species. Moreover, a relation between an average particle number and relative fluctuations derived for two-body reactions is found to be obeyed here as well despite couplings to additional equilibrium reactions in the system. Analyses of transfer reactions, where association and dissociation events take place on both sides of the chemical equation, further indicate the necessity to include cross-correlations in the expression of the equilibrium constant. However, in this case, the magnitudes of discrepancies of the uncorrelated expression are smaller, likely because of partial cancellation of correlations, which exist on both the reactant and product sides.

## Introduction

One of the most powerful postulations in chemistry is the ability to assign a constant, albeit temperature-dependent, to a chemical reaction from which the system’s composition at equilibrium can be determined by, for example, amounts of reactants put in. This equilibrium constant, *K*, is normally defined as the ratio between concentrations (activities) of products over reactants, each of which is raised to the power of its stoichiometric coefficient. That is, for the reaction
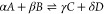
1the equilibrium constant takes the form
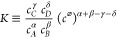
2where each concentration of each component, *c*_*X*_, is divided by a reference concentration *c*^⌀^ to render the ratio dimensionless. Alternatively, *K* is defined by
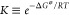
3where Δ*G*^⌀^ is the standard Gibbs free energy change when α moles of *A* react with β moles of *B* to form γ moles of *C* and δ moles of *D*, given all components are at their standard conditions. These two definitions for *K* are usually presented in textbooks together without giving importance which has precedence.^1,2^ The reason is that if we assume the chemical potential of each component, relative to that at standard state, is proportional to the logarithm of its concentration
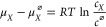
4and apply the condition of equilibrium Δ*G* = 0, these two definitions of *K* are equivalent. However, the relation in eq 4 is not always valid. Although it can be derived for a mixture of noninteracting ideal gases and observed for macroscopic systems, it is ill-defined for chemical reactions with small numbers of molecules. In these cases, the system is subject to substantial fluctuations in composition, where configurations with *c*_*X*_ = 0 are possible, and therefore, an ensemble average of μ_*X*_ cannot be properly defined. To avoid these singularities, one can define ⟨μ_*X*_⟩ by eq 4 utilizing the ensemble average of the concentration, ⟨*c*_*X*_⟩. Then, given the definition in eq 3, the expression of *K* that follows is the same as in eq 2, where the concentration of each component is an ensemble average of that concentration. It turns out, the invalidity of eq 4 is not only at singular points of *c*_*X*_ = 0 but whenever the *X* component in finite systems participates in a two-body (or higher order) reaction. As a consequence, the system’s properties, such as concentrations, are not homogeneous functions, as observed in experiments^3−11^ and simulations,^12−16^ investigating binding processes with small numbers of molecules. Furthermore, for small systems, the conventional expression of *K* (eq 2) is not constant upon changes in volumes and/or numbers of particles,^17−23^ abolishing its importance in predicting the properties of chemical reactions. On the other hand, by definition, the value of *K* in eq 3 is constant, however, taken on its own, this definition is of very limited use because it does not provide any information on reactions conducted at different concentrations or system sizes.

Recently, we utilized the definition of *K* in eq 3 to derive an equivalent expression in terms of ensemble averages of concentrations, applicable also for reactions at different conditions. To this end, a simple, elementary, two-body reaction
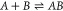
5is considered. It is found that *K* is not given by the expression widely applied in the literature,^24−33^ ⟨*c*_*AB*_⟩*c*^⌀^/(⟨*c*_*A*_⟩⟨*c*_*B*_⟩), but must include cross-correlations in reactants’ concentrations, yielding the expression^34,35^
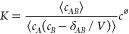
6where δ_*AB*_ equals zero for hetero-dimerizations (*A* and *B* are distinct components) and equals one for homo-dimerizations (*A* and *B* are the same components, i.e., *A* ≡ *B*). The subtraction of the reciprocal of volume term, 1/*V*, in homo-dimerizations, excludes self-correlations in particle number. For large systems, correlations between particles, as well as the term 1/*V*, can be ignored; thus, eq 6 reduces to the commonly known expression. Using different arguments, Rubinovich and Polak^36^ obtained the same expression of *K* as that shown in eq 6.

Being derived for elementary reactions, the expression of *K* for a multistep (complex) reaction, which can proceed through different mechanisms, depends on the path considered. This is because averages of correlated concentrations originating from different reaction steps cannot be canceled out in the expression of *K* describing the total complex reaction. In this paper, we study multimerizations as models for such complex reactions. It is demonstrated that despite producing different expressions for the equilibrium constant, all mechanisms (paths) yield the same value of *K*. This is true for paths composed of several two-body reactions, as well as for routes composed of elementary reaction(s) with higher-order body correlations, which, in practice, are not probable to occur. Hence, in order for a system to be in equilibrium, several relations between averages of correlated, and also uncorrelated, concentrations of different components must hold.

Throughout the manuscript, we will refer to multimerization as a process in which like-particles form a cluster larger than two and are in equilibrium with smaller clusters or monomers present at appreciable concentrations. This is to be distinguished from aggregation, in which like-particles of different cluster sizes phase-transformed abruptly to a state of one large cluster that is by far the predominant component in the system. It is interesting to note the expressions of *K* derived here produce values that stay constant also at an onset of a phase transition to an aggregated state, where concentrations of smaller-sized clusters change discontinuously.

## Results and Discussion

Consider a system at constant temperature, *T*, and volume, *V*, in which *N*_*A*_^total^ particles of *A* are able to bind with, and dissociate from, one another to form clusters of different *m*-mer sizes, *A*_*m*_, where 1 ≤ *m* ≤ *N*_*A*_^total^ (hereafter, monomers, *A*_1_, will be denoted as *A*). The behavior of all components in the system is assumed ideal, that means, except for association and dissociation events, interparticle interactions do not exist or can be ignored. All potential reactions between the *A*_*m*_ species are possible and, depending on the system investigated, quantities characterizing specific chemical equilibria can be of interest. Nevertheless, a quantity often sought for is the equilibrium constant, *K*_*m*_, of the multimerization reaction
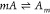
7where *m* monomers of *A* associate to form an *m*-mer, from which the corresponding free energy can be obtained. The reaction in eq 7 is not necessarily elementary, i.e., does not proceed via single step. More specifically, for *m* ≥ 3, there are different chemical paths to realize the formation of the *A*_*m*_ product, however, the most probable are likely those which require the lowest-order correlations between particles. For associations, this means sequences of two-body reactions. This is because concerted reactions of three or more particles are not likely to occur, especially in the ideal-gas approximation (low concentrations), due to the small probability to find all *m* particles simultaneously next to one another.

**Figure 1:**
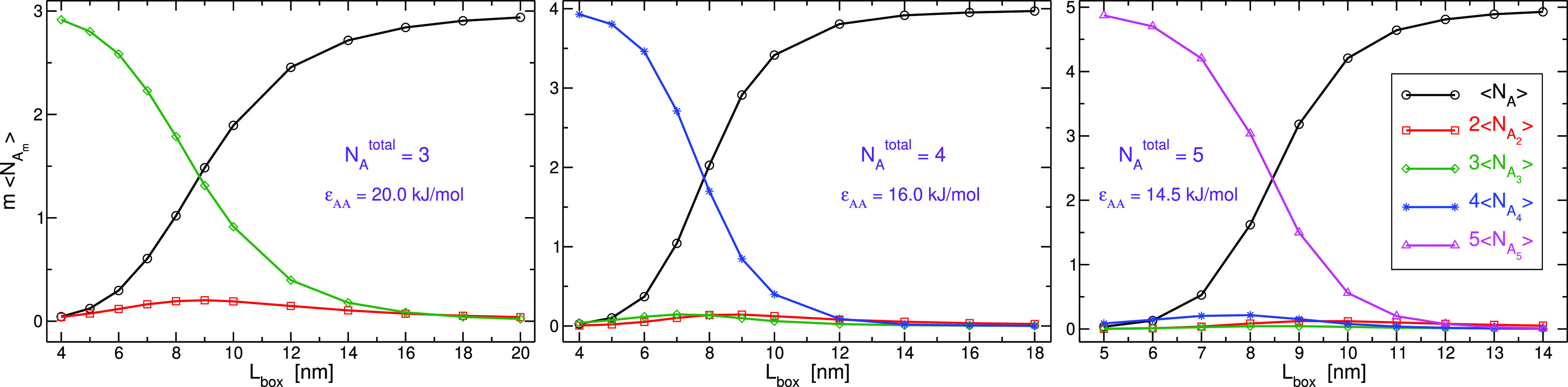
Average number of *m*-mers multiplied by *m* as a function of length of the cubic simulation box for systems with *N*_*A*_^total^ = 3, 4, and 5 of R1 series of simulations. The well-depth value of the Lennard-Jones potential acting between the particles, ϵ_*AA*_, is indicated for each system.

In the first series of simulations, R1, we considered three systems, *N*_*A*_^total^ = 3, 4, and 5, at various concentrations. The pair interaction energy between *A* particles, in each of these three systems, was chosen to support an almost complete transformation from an *A*_*m*_ state, where *m* = *N*_*A*_^total^, to an all-monomeric state upon augmenting *V* within a desired range (see the Computational Details section for more information). This behavior of the systems is shown in Figure 1 by plotting the compositions at equilibrium as a function of length of the cubic simulation box, *L*_box_.

### Two-Body Reaction Paths

**Figure 2:**
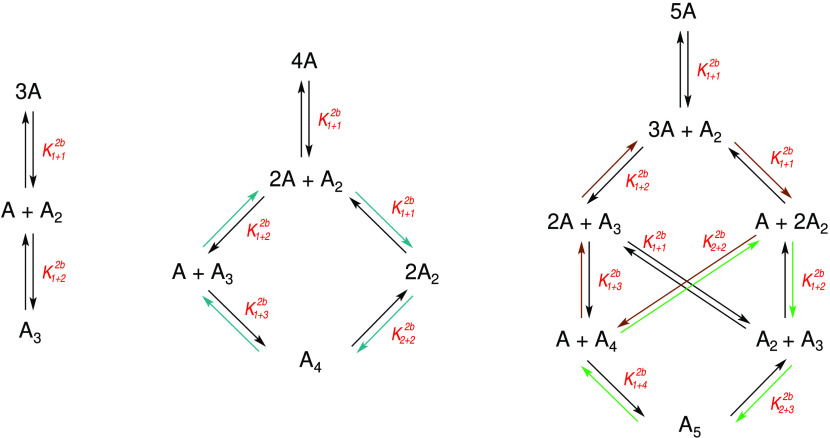
Chemical equilibria, assuming two-body-reactions, for the *N*_*A*_^total^ = 3, 4, and 5 systems (left, middle, and right panels). The two-body binding constants are indicated in red and correspond to the net chemical equations.

Chemical equilibria of all possible elementary two-body reactions are shown in Figure 2 for systems with *N*_*A*_^total^ = 3, 4, and 5, which, among others, specify the different paths connecting reactants and products of the multimerization described in eq 7. For a two-body binding reaction between an *i*-mer and a *j*-mer to form a (*i* + *j*)-mer
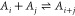
8we define the corresponding two-body binding constant *K*_*i*+*j*_^2*b*^, which equals
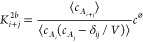
9where δ_*ij*_ is the Kronecker delta (i.e., it equals 1 if *i* = *j* and 0 otherwise). These bimolecular association constants are marked in Figure 2 and refer to the net specified equilibrium reactions. Because free energy changes are state functions, not all of the *K*_*i*+*j*_^2*b*^’s are independent. Thus, there is a smaller, irreducible, set of *K*_*i*+*j*_^2*b*^’s (whose size depends on *m*) from which the other *K*_*i*+*j*_^2*b*^’s can be derived from. It is also to be noted that the not drawn horizontal equilibria in Figure 2 for *N*_*A*_^total^ = 4 and 5 are transfer (or exchange) reactions and will be discussed below.

**Figure 3:**
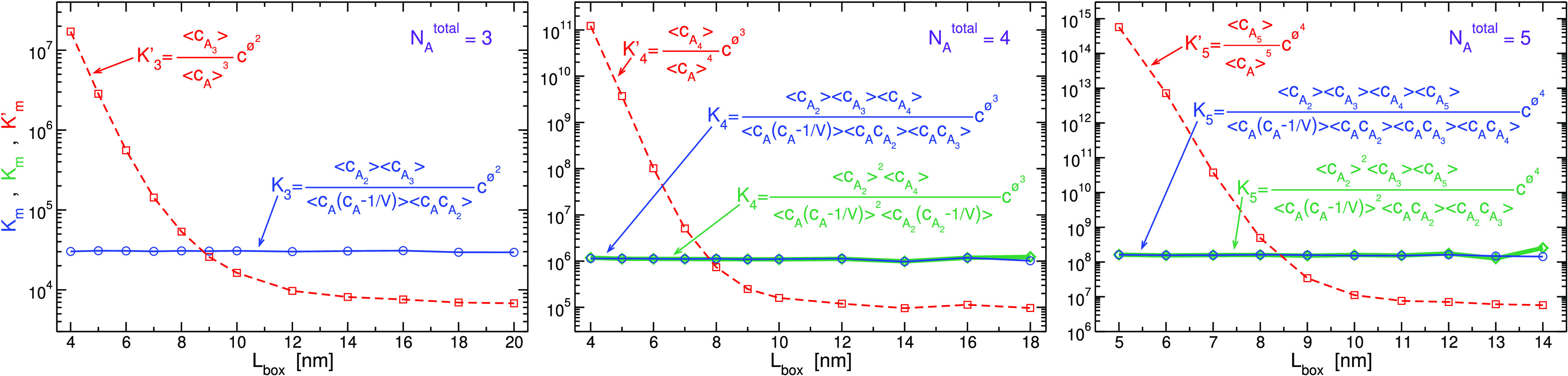
Equilibrium constant of *m*-mer formation from *m* monomers, *K*_*m*_, for *m* = 3 (trimer), 4 (tetramer), and 5 (pentamer) considering a sequence of bimolecular reactions for the three systems shown in Figure 1, respectively. Blue lines (circle symbols) are constructed by successive growths of the cluster by one monomer (eq 10). For *N*_*A*_^total^ = 4 and 5, there are additional independent routes of two-body reactions; therefore, in green lines (diamond symbols), we present also, *K*_4_ = (*K*_1+1_^2*b*^)^2^*K*_2+2_^2*b*^ and *K*_5_ = (*K*_1+1_^2*b*^)^2^*K*_1+2_^2*b*^*K*_2+3_^2*b*^, which correspond to the paths on the right side in Figure 2. For comparison, the values of the corresponding uncorrelated expressions, *K*_*m*_^′^ defined in eq 12, are shown by dashed red lines (square symbols).

The equilibrium constant of eq 7, *K*_*m*_, obtained by sequences of bimolecular reactions sketched in Figure 2 are displayed in Figure 3 for the three systems. In all cases, the resulting *K*_*m*_ is constant, independent of concentration, as it should be. For *N*_*A*_^total^ = 4 and 5, there is more than one path to form a tetramer and a pentamer, respectively. For example, the left route in Figure 2, describing successive two-body additions of monomers, leads to the following equilibrium constant
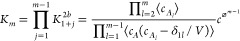
10which is different than that obtained from the route on the right. Different reaction paths produce different expressions for *K*_*m*_, some of which with no trivial dependency. However, because free energy changes are state functions, the different expressions for *K*_*m*_ must have the same value. As exhibited in Figure 3, this is indeed the case, a property which imposes dependencies on the distribution of species in the system. For example, the concentrations of, and second-order correlations between, monomer, dimer, and trimer obey the relation
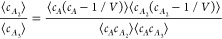
11which becomes trivial for large systems, where the terms 1/*V* and correlations can be ignored. In Figure 3, the commonly applied expression for equilibrium constant, *K*_*m*_^′^, which ignores all correlations between particles
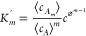
12is also shown. In contrast to the behavior of *K*_*m*_ evaluated by any path, *K*_*m*_^′^ is not constant as a function of concentration, especially in a regime of small volumes when clustering is substantial and the discrepancies reach several orders of magnitude. Even at large volumes, when it becomes constant, *K*_*m*_^′^ does not approach *K*_*m*_ because self-correlations are significant for systems with small values of *N*_*A*_^total^. For completeness, in Figure SI-1 of the Supporting Information (SI), we present the two-body binding constants of reactions with at least one monomer, *K*_1+*j*_^2*b*^. Also here, all equilibrium constants are constant for all values of *L*_box_, whereas the corresponding expressions not accounting for two-body correlations between particles, *K*_1+*j*_^′2*b*^, are not. Furthermore, the discrepancy of *K*_1+*j*_^′2*b*^ from *K*_1+*j*_^2*b*^ at large volumes decreases with *N*_*A*_^total^ and increases with *j*.

**Figure 4:**
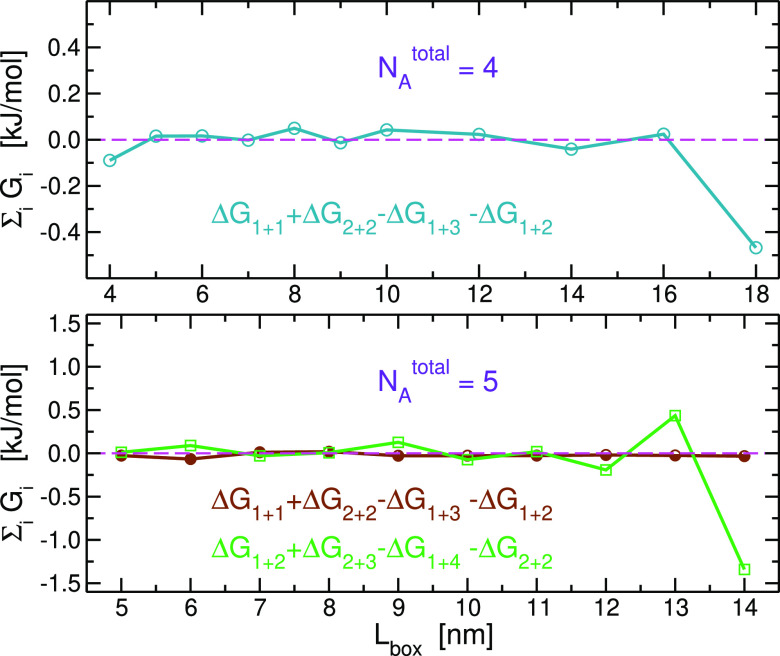
Thermodynamic cycle closures of the two-body-reaction model for *N*_*A*_^total^ = 4 and 5 of R1 series of simulations. For the former, there is only one independent cycle involving two-body association constants, whereas, for the latter, there are two independent cycles. The cycles are depicted in Figure 2 with colors of the arrows matching the colors of the curves here. The free energy changes are defined by Δ*G*_*i*+*j*_ = −*RT* ln *K*_*i*+*j*_^2*b*^. The dashed magenta lines at *y* = 0 denote perfect closure.

The fact that not all *K*_*i*+*j*_^2*b*^’s are independent can also be illustrated by constructions of thermodynamic cycles, where the sum of free energy changes in a closed cycle should be equal to zero. The magnitude of deviation from zero is an estimate to error in the computation. When applied to our systems, as shown in Figure 4, the results indicate that the deviations are smaller than 0.2 kJ/mol except for the largest two *L*_box_ values, where they reach 1.3 kJ/mol in closing one cycle of the *N*_*A*_^total^ = 5 system. Obviously, obtaining converged results is more difficult for systems with a larger volume and number of particles.

### Transfer Reactions

**Figure 5:**
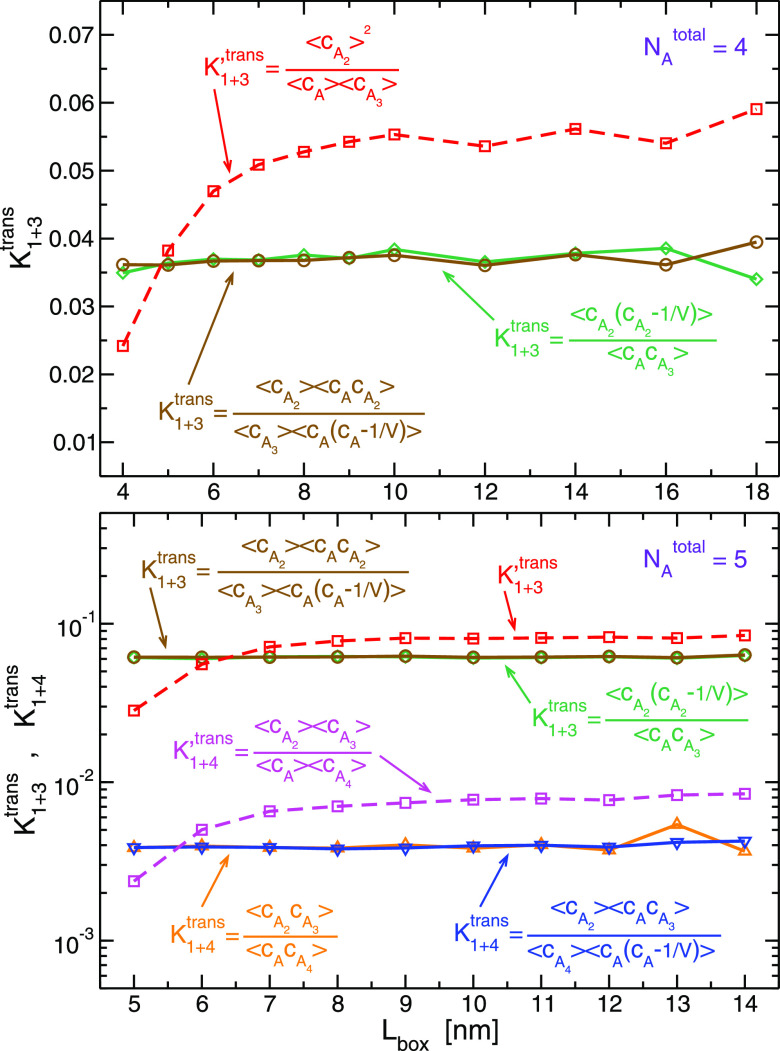
Equilibrium constants, *K*_1+3_^trans^ and *K*_1+4_^trans^, for the transfer reactions, *A* + *A*_3_ ⇌ 2*A*_2_ and *A* + *A*_4_ ⇌ *A*_2_ + *A*_3_, respectively, of the *N*_*A*_^total^ = 4 and 5 systems. Green (diamonds) and orange (triangles pointing up) curves were calculated by eq 14, whereas brown (circles) and blue (triangles pointing down) were calculated by eq 15. The corresponding expressions ignoring correlations, *K*_1+3_^′trans^ and *K*_1+4_^′trans^, are presented by dashed lines (squares) in red and magenta, respectively.

As mentioned above, the unmarked horizontal reactions in Figure 2 of the type
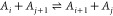
13correspond to transfer reactions, where in both forward and backward directions, association and dissociation occur. Taking cross-correlations into account, the expression of the equilibrium constant is given by
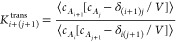
14where the standard concentration, *c*^⌀^, cancels out because the number of particles, thus phase space, is the same on both sides of the chemical equation. Alternatively, the same reaction can be described by subtracting a two-body binding reaction, *A*_*j*_ + *A* ⇌ *A*_*j*+1_, from another two-body binding reaction, *A*_*i*_ + *A* ⇌ *A*_*i*+1_, and consequently its equilibrium constant equals
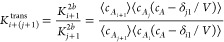
15Clearly, the expressions in eqs 14 and 15 must be equal and independent of concentration. As shown in Figure 5, such is the case for the three unmarked transfer reactions of the equilibrium schemes in Figure 2. Again discrepancies, which are small in magnitude, appear only at large volumes. Furthermore, the corresponding uncorrelated expressions
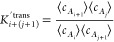
16are not constant, especially at small values of *L*_box_. It should be noted though, their deviations from the value of the equilibrium constant (either eqs 14 or 15) are much smaller, less than an order of magnitude, than for binding reactions (see Figures 3 and SI-1), which can reach several orders of magnitudes. It is likely that cross-correlations, present on both sides of transfer reactions (eq 13), partially cancel each other.

### Paths Involving Three-, or Higher-Order, Body Reactions

**Figure 6:**
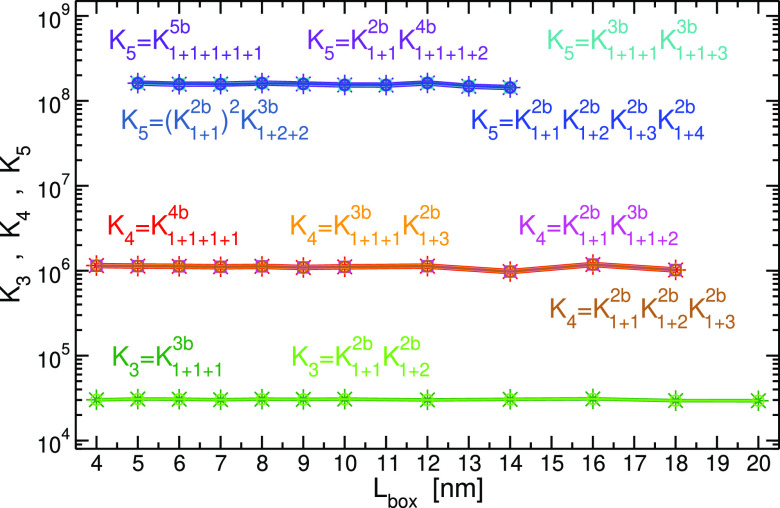
Equilibrium constant for *m*-mer formation, *K*_*m*_, the same as that calculated in Figure 3 but here considering paths involving higher-than-two-body reactions. As before, *K*_3_, *K*_4_, and *K*_5_ are calculated from systems with *N*_*A*_^total^ = 3, 4, and 5, respectively. The results of different order body reactions are very similar, and as references, we also show those obtained by the two-body reaction route of gradual monomer additions, *K*_*m*_ = ∏_*j*=1_^*j* = *m*–1^*K*_1+*j*_^2*b*^ (eq 10), presented in Figure 3.

We argued above that three-, or higher-order, body reactions are not likely to occur for reactants with unrestricted motions at low concentrations. Still, due to the state function character of a change in free energy, it should be possible to compute equilibrium constants by these implausible paths. Even if these reactions would practically never proceed via these mechanisms, the probabilities of observing the states on both sides of the chemical equation (each in equilibrium with other viable reaction mechanisms) do permit to calculate the equilibrium constant. Here, we show that these calculations yield results equal to those obtained by any other possible path. We start by writing the expression of *K*_*m*_ for the multimerization reaction described in eq 7 for the case when all m monomers react simultaneously, that is, when eq 7 describes an elementary reaction. Because this *m*-body reaction is concerted, cross-correlations in reactant concentrations should include (*m* – 1) successive subtractions of self-correlations, and therefore, the *m*-body equilibrium constant takes the form
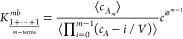
17where the summation label of *m*-terms of 1’s in the subscript of *K* indicates a simultaneous reaction between *m* monomers. The same expression was obtained by Rubinovich and Polak,^36^ however, without the claim, it corresponds to a concerted *m*-body reaction. Definitely, other *n*-order body reactions (3 ≤ *n* < *N*_*A*_^total^ – 1) involving clusters of larger size are also possible, where the expressions of the corresponding equilibrium constants can be easily inferred. For example, the equilibrium constant of a three-body reaction between two monomers and a dimer to form a tetramer is *K*_1+1+2_^3*b*^ = ⟨*c*_*A*_4__⟩*c*^⌀^2^^/⟨*c*_*A*_(*c*_*A*_ – 1/*V*)*c*_*A*_2__⟩. We compute *K*_*m*_ for *m* = 3, 4, and 5 in the systems *N*_*A*_^total^ = 3, 4, and 5, respectively, using all possible *n*-order body reactions and present the results in Figure 6. To eliminate ambiguity, we specify the mechanisms by outlining the elementary reactions involved and the total expressions of *K*_*m*_ in Table SI-1. Figure 6 demonstrates that all possible mechanisms, including those with different orders of body reactions, of the multimerization of eq 7 yield the same value of *K*_*m*_. Here, again, different expressions representing the same quantity of *K*_*m*_ establish relations between averages of correlated, or uncorrelated, concentrations of different species in the system. For example, equating eq 10 with eq 17 results in
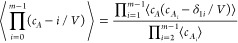
18

We note, even though all different paths (mechanisms) produce the same value for the equilibrium constant, they would not yield the same value for the corresponding reaction rates. Again, some (higher-order body reaction) mechanics are not likely to occur at all.

### Multimerizations in Systems with Different *N*_*A*_^total^

**Figure 7:**
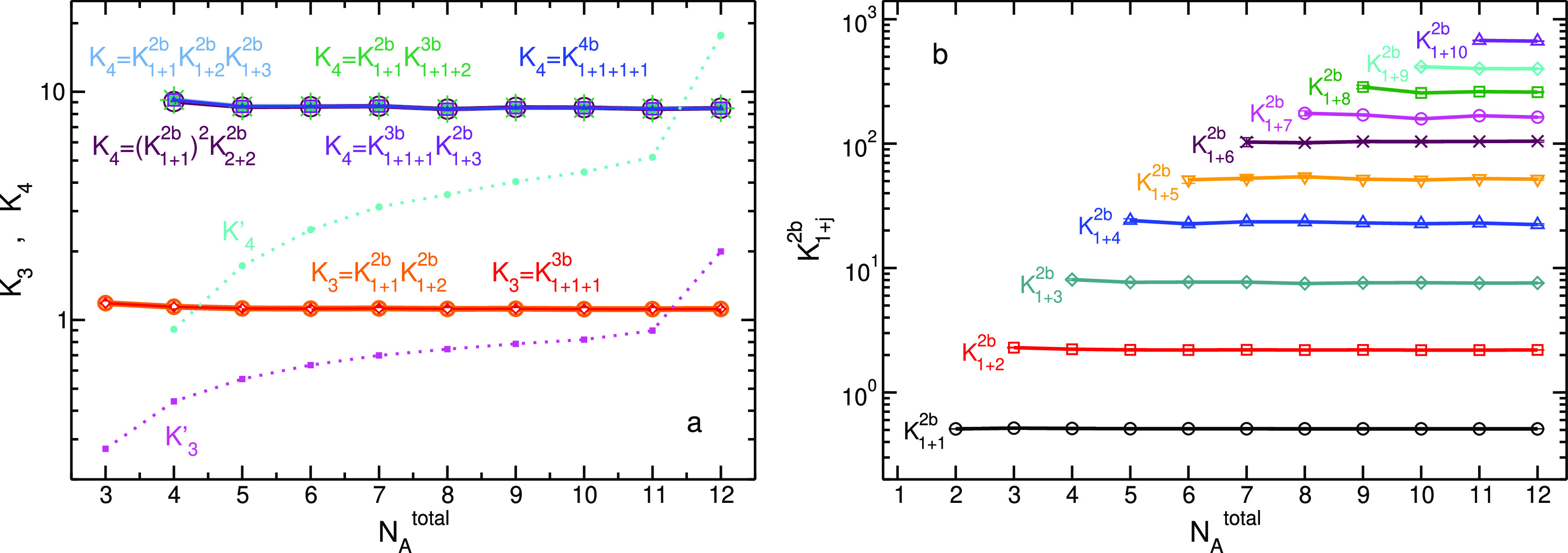
Results from R2 series of simulations (where the association energy between monomers, ϵ_*AA*_ = 10.0 kJ/mol, is the same for all systems). (a) Equilibrium constants for trimer (*K*_3_) and tetramer (*K*_4_) formation from the corresponding number of monomers as a function of *N*_*A*_^total^. The value of *K*_3_ is calculated by a product of two two-body reactions (*K*_1+1_^2*b*^*K*_1+2_^2*b*^) as well as by a three-body reaction (*K*_1+1+1_^3*b*^). *K*_4_ is calculated in five different ways: by a product of three two-body reactions [*K*_1+1_^2*b*^*K*_1+2_^2*b*^*K*_1+3_^2*b*^ and (*K*_1+1_^2*b*^)^2^*K*_2+2_^2*b*^], by a product of two-body and three-body reactions (*K*_1+1_^2*b*^*K*_1+1+2_^3*b*^ and *K*_1+1+1_^3*b*^*K*_1+3_^2*b*^), and by a four-body reaction (*K*_1+1+1+1_^4*b*^). See Table SI-1 of SI for explicit expressions of all equilibrium constants. We also present as dotted lines the value of the expressions ignoring correlations, *K*_3_^′^ (magenta) and *K*_4_^′^ (cyan), as defined in eq 12. (b) Equilibrium constants of two-body reactions involving a monomer, *K*_1+*j*_^2*b*^. In both plots, the magnitudes of the estimated errors are smaller or similar to the size of symbols.

In the analyses above, we did not attempt to compare values of *K*_*m*_ extracted from systems with different total numbers of monomers. The reason is because the well-depth values of the interaction energy, ϵ_*AA*_, are different (resulting from the aim to get continuous transformations between a predominantly monomeric state and a predominantly aggregated state within a certain range of *L*_box_ as exhibited in Figure 1), and therefore, the values of *K*_*m*_, for the same *m*, across different systems are different as well. To rectify this situation, we conducted a second series of simulations, R2, where the binding potential is the same for all systems, ϵ_*AA*_ = 10 kJ/mol, however, the total number of monomers was changed in the range of 2 ≤ *N*_*A*_^total^ ≤ 12, keeping its total concentration constant (0.03245 *M*). In Figure 7a, we plot the value of *K*_3_ and *K*_4_ extracted from systems with different *N*_*A*_^total^. *K*_3_ is calculated by two mechanisms, one involving two-body and the other three-body reactions. *K*_4_ is computed by five expressions, representing paths of two-, three-, and four-body reactions. The results indicate all different mechanisms, which lead to different expressions of *K*_*m*_, give the same value, and are invariant of system size. The same conclusion is obtained by plotting the two-body equilibrium constants *K*_1+*j*_^2*b*^, defined by eq 9 for *i* = 1, in Figure 7b. The significance of these results is that the equilibrium constant does not depend on whether the reaction in question is the only reaction taking place in the system or on whether the reactant and/or product engage with many other equilibrium reactions involving foreign compounds. This is obvious from the definition of the equilibrium constant in eq 3 and is illustrated, for example, by *K*_1+1_^2*b*^ (≡*K*_2_) for *N*_*A*_^total^ = 2, where dimerization of monomers and dissociation of dimers are the only two possible processes in the system, and for *N*_*A*_^total^ = 12, where the reactant and product take part in 10 and 9, respectively, additional equilibrium reactions.

In Figure 7a, we also plot the values of *K*_3_^′^ and *K*_4_^′^, the expressions ignoring correlations in the systems as defined in eq 12. As expected, large deviations are observed for small *N*_*A*_^total^, which decrease with an increase in a total number of monomers. However, at approximately *N*_*A*_^total^ = 11, there is an abrupt increase in the values of *K*_3_^′^ and *K*_4_^′^. Analyses of the concentrations of monomers, trimers, and tetramers (Figure SI-2a,b) indicate a sharp decrease exactly around this value of *N*_*A*_^total^ for these three species, and as a result, the curves of *K*_3_^′^ and *K*_4_^′^ experience a discontinuous increase because the monomer concentration is raised to a power of 3 and 4 in the denominator. These sharp drops in concentrations are most likely due to an onset of a phase transition to an aggregated state. Indeed, there is an increase in the concentration of the largest possible cluster in the system (Figure SI-2c) at that point, however, apparently with a continuous character. It is worth emphasizing that this onset of phase transition (aggregation), although drastically affects *K*_3_^′^ and *K*_4_^′^, does not influence the values of *K*_3_ and *K*_4_, which stay constant also across this point.

### Relative Fluctuations in Particle Numbers

**Figure 8:**
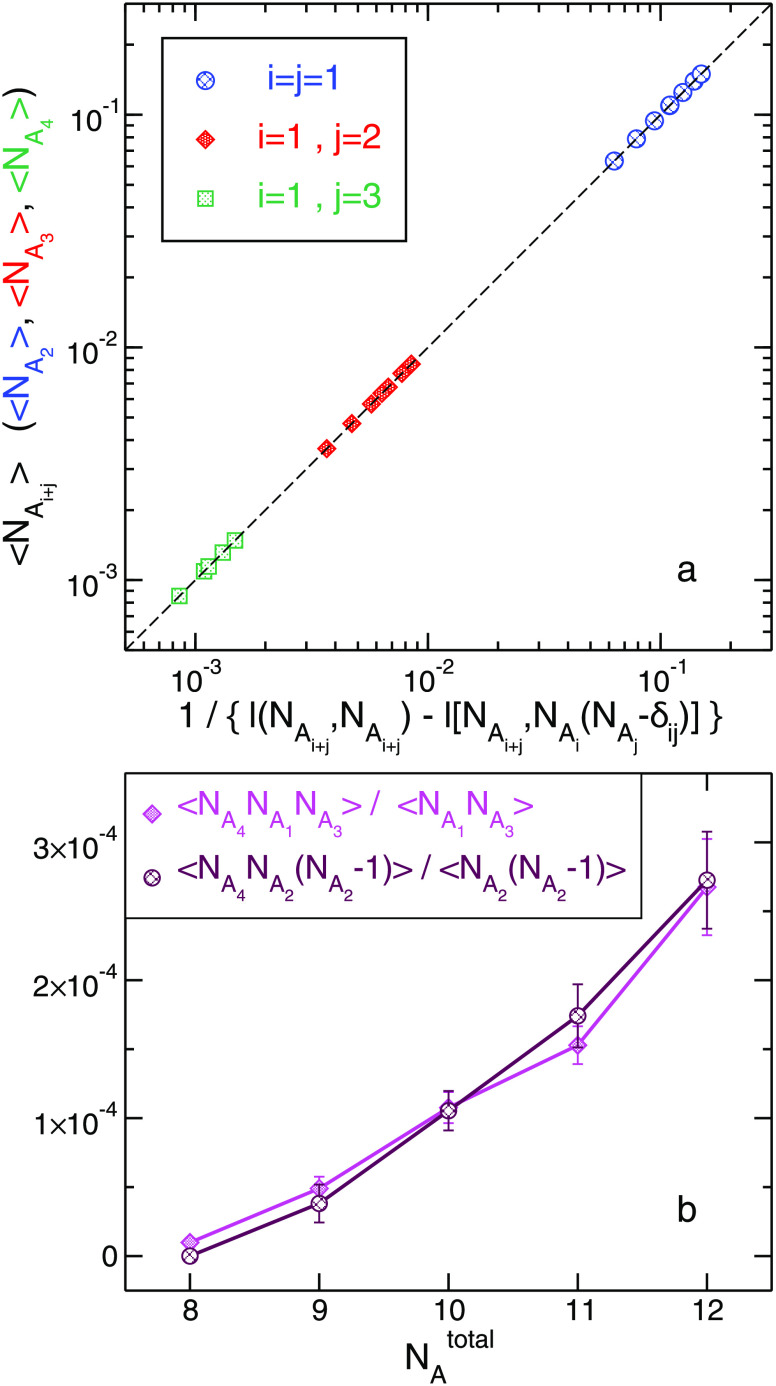
(a) Relation between the average number of dimer (blue), trimer (red), and tetramer (green) and relative fluctuations in the system as described by eq 19. The results are obtained from R2 series for systems with *N*_*A*_^total^ ≥ 5, 6, and 8 for dimer, trimer, and tetramer, respectively. The dashed black line, *y* = *x*, is plotted as a reference. (b) Validation of the equality described in eq 20 for the case *i* + *j* = *k* + *s* = 4 (*i* = 1, *j* = 3, and *k* = *s* = 2) for simulations with 8, or larger, number of total *A* particles.

Previously, we showed that for homo- and hetero-dimerizations, the average number of bound particles is related to relative fluctuations in the system.^34,35^ Accordingly, for the two-body reaction described in eq 8, we can write the average number of a cluster of size (*i* + *j*) as
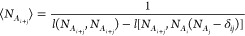
19where the relative fluctuations between two variables ζ and η are defined by *l*(ζ, η) = ⟨ζη⟩/(⟨ζ⟩⟨η⟩) – 1. In Figure 8a, we test this equality on R2 series for systems with *N*_*A*_^total^ ≥ 5, 6, and 8 for dimer, trimer, and tetramer, respectively. This is because the relation is trivial for systems with a smaller number of particles (which includes the entire R1 series). The results obtained exhibit an almost perfect agreement.

For systems with *N*_*A*_^total^ ≥ 4, the sum (*i* + *j*) can be decomposed into different sets of integers (not including zero). If *i* + *j* = *k* + *s*, then equating the expression of ⟨*N*_*A*_*i*+*j*__⟩ with that of ⟨*N*_*A*_*k*+*s*__⟩ (both using eq 19) yields the relative fluctuations *l*[*N*_*A*_*i*+*j*__, *N*_*A*_*i*__(*N*_*A*_*j*__ – δ_*ij*_)] equal the relative fluctuations *l*[*N*_*A*_*k*+*s*__, *N*_*A*_*k*__(*N*_*A*_*s*__ – δ_*ks*_)], or more explicitly
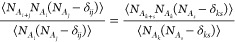
20This relation is examined in Figure 8b for *N*_*A*_^total^ = 4 of R2 series of simulations. Although some discrepancies are noticeable, only for the smallest system, the agreement falls just outside the estimated error bars.

### Calculating *K*_*m*_ from the Ratio of Probabilities of Occurrences

Examining the expression of *K*_*m*_ derived from the *m*-body reaction, eq 17, it is easy to show that for the private case of *N*_*A*_^total^ = *m*, this expression can also be written as the ratio of probability to observe the system in the *m*-mer state (*A*_*m*_) to probability to observe all *mA* particles as monomers. More explicitly, if *f*^*A*_*m*_^ and *f*^*mA*^ are fractions of frames (or probabilities) in which all particles are clustered and in which all particles are monomers, respectively, *K*_*m*_ takes the form
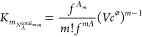
21which is equivalent to eq 17 and corresponds to expressions obtained by Ouldridge and co-workers for dimerization^37^ and hexamer formation^38^ for the private case discussed here.

Last, and likely least, we inspect again the densities of different species of R1 series of simulations presented in Figure 1. It is evident the predominant components at equilibrium are either monomer or largest-mer possible, and hence, it might be tempting to approximate the behavior of these systems by a two-state model. This means densities of other species (states) are ignored, and it is enough to determine the fraction of frames of only one state (*f*^*A*_*m*_^ or *f*^*mA*^), where the fraction of frames of the second state is obtained by subtraction from one. Assessing this approximation (Figure SI-3) reveals very poor agreement with the exact results of eq 21. If we choose to determine *f*^*A*_*m*_^ (*f*^*mA*^), it is only for a regime in which the state of *A*_*m*_ (*mA* monomers) is hardly observed that *K*_*m*_ is adequately approximated.

## Conclusions

For large systems, the expression of equilibrium constant for a complex reaction, which can proceed via several alternate mechanisms, is known to be independent of the mechanism considered. This is because cross-correlations between particle numbers are negligible, and therefore, average concentrations originating from different reaction steps can be canceled out in nominator and denominator of the total equilibrium constant, leaving only those corresponding to the net reaction. However, for finite systems, cross-correlations must be taken into account and cancellations of concentrations are, in general, not possible. This means, for each reaction mechanism (path), there is a unique expression of the equilibrium constant. At the same time, equilibrium constants are defined by free energy changes and as state functions, their values must be path independent. In this paper, we showed that different expressions of the equilibrium constant for a multimer formation, arising from different routes, all yield the same value. Thus, equalities between the different expressions characterize equilibrium, which translates to equalities between averages of concentrations (correlated or uncorrelated). Put another way, the condition for equilibrium includes additional restrictions on the distribution of particle numbers in the systems. At first glance, it might appear tantalizing that an expression of equilibrium constant corresponding to a path not likely to occur, such as a concerted approach of five monomers to form a pentamer, would give the same value as those derived from path(s) that do happen. However, this is indeed the case, and reaction routes which do not take place can also be utilized for calculating *K* as long as there is at least one physically viable path connecting reactants with products.

Another characteristic of equilibrium constant demonstrated in this work is that its value is constant for all system sizes (down to the smallest system possible) and concentrations, as should be the case. In contrast, the commonly utilized expression applicable for the thermodynamic limit, which ignores correlations, produces values that vary significantly with changes in volume and the total number of particles. It is interesting to point out that for transfer reactions, the error of ignoring correlations is much smaller than that for corresponding association and dissociation reactions. This can be explained by partial cancellation of correlations, which appear on both sides of the chemical equation of transfer reactions.

For hetero- and homo-dimerizations, magnitudes of relative fluctuations are related to average concentrations. This equality is found to be valid also here, thus for larger-sized cluster formations, where reactants and products are coupled to other equilibrium reactions. When considering clusters of sizes equal to or larger than four, the different ways to partition the integer representing the cluster size into sum of smaller integers establish relations between correlated averages of different particle numbers in the system (eq 20).

## Computational Details

The model system consists of *N*_*A*_^total^ single-site *A* particles interacting solely via Lennard-Jones (LJ) potential with a diameter σ_*AA*_ = 0.20 nm and a well-depth ϵ_*AA*_. Newton’s equations of motion were applied to propagate the system in time in such a way the resulting trajectory generated a canonical ensemble (*N*_*A*_^total^, *V*, *T*) at a temperature *T* = 300 K and a volume *V* = *L*_box_^3^, with *L*_box_ the length of the cubic simulation box.

Two series of simulations were designed. In the first, R1, we studied systems with *N*_*A*_^total^ = 3, 4, and 5, and modified systematically *L*_box_. For each *N*_*A*_^total^, the value of ϵ_*AA*_ was chosen such that within the range of *L*_box_ considered, an almost complete transformation occurred from a monomeric state to an aggregated state (see Figure 1). The values of ϵ_*AA*_, together with the range of *L*_box_ investigated, are presented in Table 1. In the second series of simulations, R2, the well-depth of the interactions between the particles was constant, ϵ_*AA*_ = 10 kJ/mol, and we changed the total number of *A* particle in the system in the range 2 ≤ *N*_*A*_^total^ ≤ 12 and concomitantly *L*_box_, such that the concentration *N*_*A*_^total^/*V* equals 0.03245 *M* (i.e., 0.01954 molecules/nm^3^). This resulted in a box length of 4.678 and 8.500 nm for the smallest and largest systems, respectively.

**Table 1:** Well-Depth of Lennard-Jones Potential and Range of Lengths of Cubic Simulation Boxes for the Three Systems of R1

*N*_*A*_^total^	ϵ_AA_ [kJ/mol]	*L*_box_ [nm]
3	20.0	4.0–20.0
4	16.0	4.0–18.0
5	14.5	5.0–14.0

Molecular dynamics (MD) simulations were conducted by the software package GROMACS version 4.6.5^39^ (single-precision). A time step of 0.002 ps was employed for integrating the equations of motion and a mass of 10.0 amu was assigned to each *A* particle. The temperature was maintained by applying a Nosé–Hoover thermostat^40,41^ with a chain length^42^ of 2 and a coupling strength set to 0.1. The equations of motion were propagated by the velocity Verlet algorithm, in which the kinetic energy is determined by the average of the two half-steps. Periodic boundary conditions were applied along all three Cartesian axes, and at every step, motion of center of mass of the system was removed. The LJ potential was evaluated up to a cutoff distance of 2.0 nm. Based on the location of the first minimum of the radial distribution function, two *A* particles are defined as clustered (bonded) for *r*_*AA*_ < 0.35 nm.

For all simulations, equilibration for approximately 5 μ*s* was conducted prior to data collection. In R1 series for *N*_*A*_^total^ = 3 and 4, at each value of *L*_box_, data were collected for 400 μs. However, for *N*_*A*_^total^ = 4 with *L*_box_ ≥ 14 and for *N*_*A*_^total^ = 5, the data collection time was doubled, thus for 800 μs. In R2 series, the data collection step for each *N*_*A*_^total^ was run for 1600 μs.

## Data Availability

Input (parameter, configuration, and topology) files of R1 and R2 series of simulations necessary to reproduce all data presented in this paper are available at https://zenodo.org/record/7956480#.ZGs3vxlBzmF. Gromacs version 4.6.5, used for all simulations in this work, is available to the public via the link https://manual.gromacs.org.

## Supporting Information Available

(1) Figure of the set of *K*_1+*j*_^2*b*^, (2) table specifying different expressions to calculate *K*_*m*_, (3) figure of average concentrations in R2 series of simulations, and (4) figure of *K*_*m*_ approximated by a two-state model (PDF)
