# Supplementary material for: Multimerizations, Aggregation, and Transfer Reactions of Small Numbers of Molecules
Source: J Chem Inf Model. 2023 Jul 11;63(14):4383–91. doi: 10.1021/acs.jcim.3c00774 (PMC10369493; doi:10.1021/acs.jcim.3c00774)
Supplement: Supplementary file 1 — ci3c00774_si_001.pdf [file ci3c00774_si_001.pdf]

Supporting Information: Multimerizations, Aggregation, and Transfer Reactions of Small Numbers of Molecules

Ronen Zangi*[1,2,3]

[1] *Donostia International Physics Center (DIPC), 20018 Donostia-San Sebastián, Spain*

[2] *Department of Organic Chemistry I, University of the Basque Country UPV/EHU, 20018 Donostia-San Sebastián, Spain*

[3] *IKERBASQUE, Basque Foundation for Science, 48009 Bilbao, Spain*

June 22, 2023

*r.zangi@ikerbasque.org

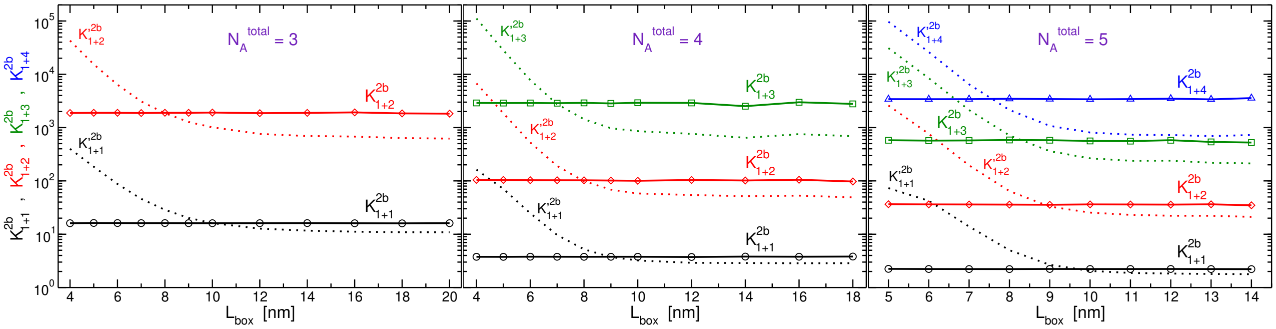

Figure SI-1: The set of equilibrium constants for two-body elementary reactions involving at least one monomer, K^{2b}_{1+j}, defined by Eq. 9 with $i = 1$, for the three systems of R1 series of simulations (solid lines with symbols). The values of the corresponding uncorrelated expressions, $K'^{2b}_{1+j} = \langle c_{A_{1+j}} \rangle c^{\varnothing} / (\langle c_A \rangle \langle c_{A_j} \rangle)$, are shown as well (dotted lines).

Table SI-1: Different expressions to calculate K_m (for $m = 3$, 4, and 5) depending on the elementary reaction(s) considered. As shown in Fig. 6, all expressions yield the same value.

K_m	highest order correlations	elementary reaction(s)	total expression of K_m
K_3	two-body	$A + A \rightleftharpoons A_2$<br>$A + A_2 \rightleftharpoons A_3$	$K^{2b}_{1+1}K^{2b}_{1+2} = \frac{\langle c_{A2}\rangle\langle c_{A3}\rangle\ {c^{\varnothing}}^2}{\langle c_A(c_A-1/V)\rangle\langle c_A c_{A2}\rangle}$
	three-body	$A + A + A \rightleftharpoons A_3$	$K^{3b}_{1+1+1} = \frac{\langle c_{A3}\rangle\ {c^{\varnothing}}^2}{\langle c_A(c_A-1/V)(c_A-2/V)\rangle}$
K_4	two-body	$A + A \rightleftharpoons A_2$<br>$A + A_2 \rightleftharpoons A_3$<br>$A + A_3 \rightleftharpoons A_4$	$K^{2b}_{1+1}K^{2b}_{1+2}K^{2b}_{1+3} = \frac{\langle c_{A2}\rangle\langle c_{A3}\rangle\langle c_{A4}\rangle\ {c^{\varnothing}}^3}{\langle c_A(c_A-1/V)\rangle\langle c_A c_{A2}\rangle\langle c_A c_{A3}\rangle}$
	three-body	$A + A + A \rightleftharpoons A_3$<br>$A + A_3 \rightleftharpoons A_4$	$K^{3b}_{1+1+1}K^{2b}_{1+3} = \frac{\langle c_{A3}\rangle\langle c_{A4}\rangle\ {c^{\varnothing}}^3}{\langle c_A(c_A-1/V)(c_A-2/V)\rangle\langle c_A c_{A3}\rangle}$
	three-body	$A + A \rightleftharpoons A_2$<br>$A + A + A_2 \rightleftharpoons A_4$	$K^{2b}_{1+1}K^{3b}_{1+1+2} = \frac{\langle c_{A2}\rangle\langle c_{A4}\rangle\ {c^{\varnothing}}^3}{\langle c_A(c_A-1/V)\rangle\langle c_A(c_A-1/V)c_{A2}\rangle}$
	four-body	$A + A + A + A \rightleftharpoons A_4$	$K^{4b}_{1+1+1+1} = \frac{\langle c_{A4}\rangle\ {c^{\varnothing}}^3}{\langle c_A(c_A-1/V)(c_A-2/V)(c_A-3/V)\rangle}$
K_5	two-body	$A + A \rightleftharpoons A_2$<br>$A + A_2 \rightleftharpoons A_3$<br>$A + A_3 \rightleftharpoons A_4$<br>$A + A_4 \rightleftharpoons A_5$	$K^{2b}_{1+1}K^{2b}_{1+2}K^{2b}_{1+3}K^{2b}_{1+4} = \frac{\langle c_{A2}\rangle\langle c_{A3}\rangle\langle c_{A4}\rangle\langle c_{A5}\rangle\ {c^{\varnothing}}^4}{\langle c_A(c_A-1/V)\rangle\langle c_A c_{A2}\rangle\langle c_A c_{A3}\rangle\langle c_A c_{A4}\rangle}$
	three-body	$A + A + A \rightleftharpoons A_3$<br>$A + A + A_3 \rightleftharpoons A_5$	$K^{3b}_{1+1+1}K^{3b}_{1+1+3} = \frac{\langle c_{A3}\rangle\langle c_{A5}\rangle\ {c^{\varnothing}}^4}{\langle c_A(c_A-1/V)(c_A-2/V)\rangle\langle c_A(c_A-1/V)c_{A3}\rangle}$
	three-body	$A + A \rightleftharpoons A_2$ x 2<br>$A + A_2 + A_2 \rightleftharpoons A_5$	$(K^{2b}_{1+1})^2K^{3b}_{1+2+2} = \frac{\langle c_{A2}\rangle^2\langle c_{A5}\rangle\ {c^{\varnothing}}^4}{\langle c_A(c_A-1/V)\rangle^2\langle c_A c_{A2}(c_{A2}-1/V)\rangle}$
	four-body	$A + A \rightleftharpoons A_2$<br>$A + A + A + A_2 \rightleftharpoons A_5$	$K^{2b}_{1+1}K^{4b}_{1+1+1+2} = \frac{\langle c_{A2}\rangle\langle c_{A5}\rangle\ {c^{\varnothing}}^4}{\langle c_A(c_A-1/V)\rangle\langle c_A(c_A-1/V)(c_A-2/V)c_{A2}\rangle}$
	five-body	$A{+}A{+}A{+}A{+}A \rightleftharpoons A_5$	$K^{5b}_{1+1+1+1+1} = \frac{\langle c_{A5}\rangle\ {c^{\varnothing}}^4}{\langle c_A(c_A-1/V)(c_A-2/V)(c_A-3/V)(c_A-4/V)\rangle}$

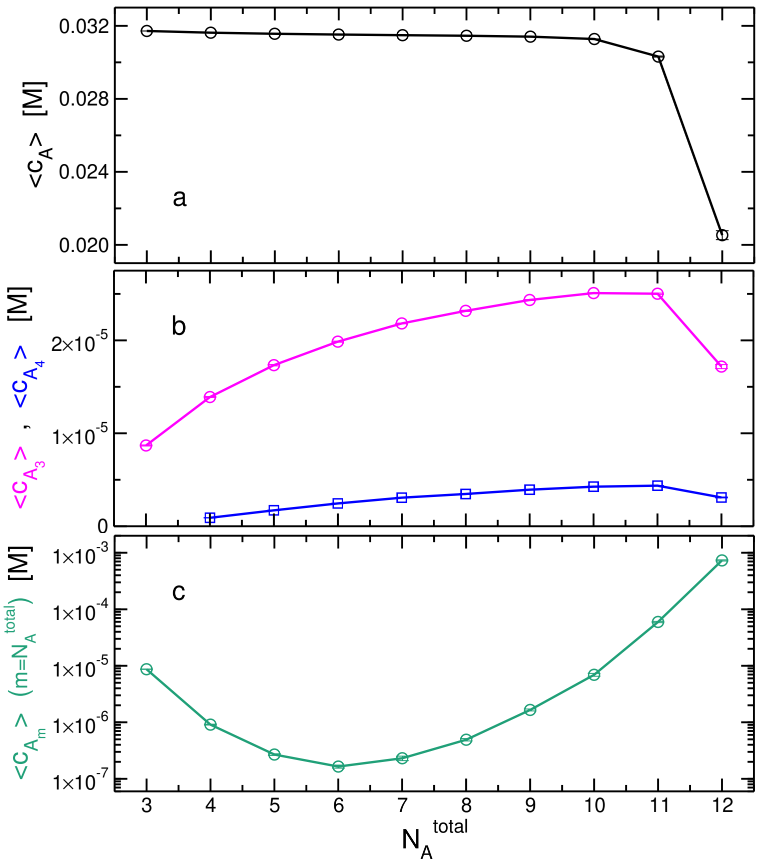

Figure SI-2: Average concentrations of several m-mers as a function of N_A^{total} for R2 series of simulations. (a) For monomer, (b) for trimer and tetramer, (c) for the largest multi-mer (cluster) possible, thus for A_m with $m = N_A^{\text{total}}$. Note that for all simulations in this series, the concentration of N_A^{total}/V is constant at $0.03245\ M$.

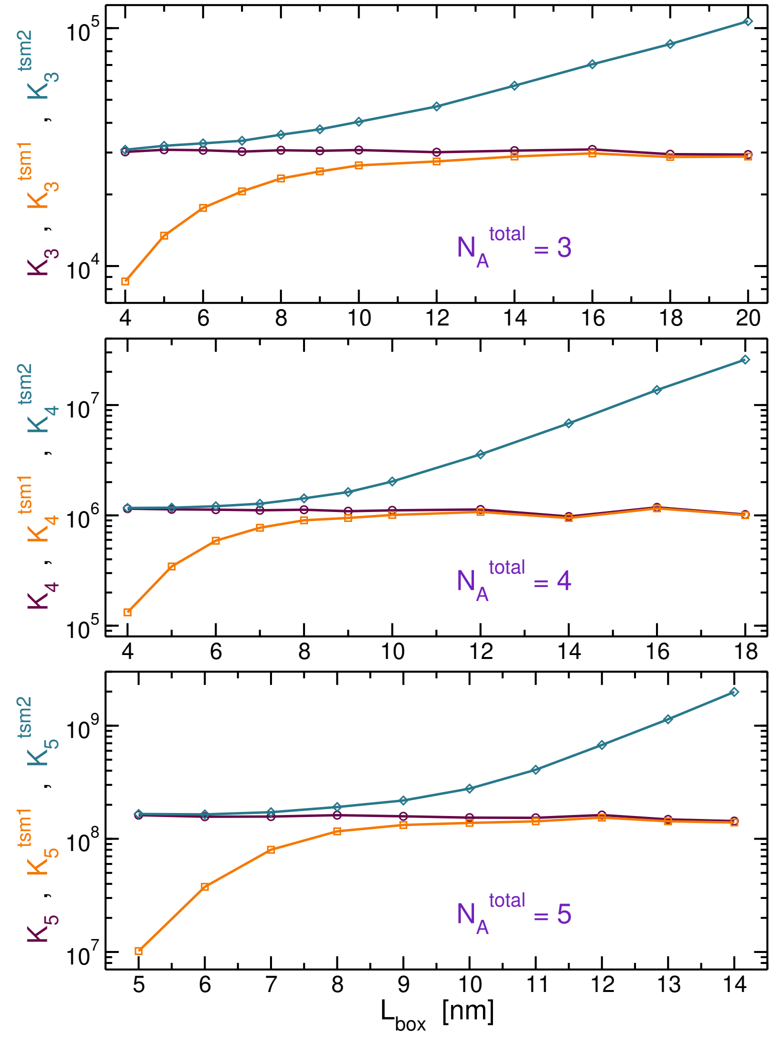

Figure SI-3: Approximations of K_m for a private case of $m = N_A^{\text{total}}$ assuming a two-state model. The curves in orange (square symbols) are calculated by $K_m^{tsm1} = f^{A_m}(Vc^{\varnothing})^{m-1}/[m!(1-f^{A_m})]$, whereas the curves in blue (diamonds) are computed by $K_m^{tsm2} = (1-f^{mA})(Vc^{\varnothing})^{m-1}/[m!f^{mA}]$, where f^{A_m} and f^{mA} are fractions of frames, or probabilities, of observing the system in A_m and mA (i.e., all m A particles are monomers) states, respectively. As references for exact results, we plot in maroon (circles) $K_m = f^{A_m}(Vc^{\varnothing})^{m-1}/[m!f^{mA}]$ (Eq. 21).
